# Long Non-Coding RNA AP000695.2 Acts as a Novel Prognostic Biomarker and Regulates the Cell Growth and Migration of Lung Adenocarcinoma

**DOI:** 10.3389/fmolb.2022.895927

**Published:** 2022-05-24

**Authors:** Chunyan Wang, Jishu Guo, Rongyan Jiang, Chenyang Wang, Chenglong Pan, Zhi Nie, Xiulin Jiang

**Affiliations:** ^1^ Department of Pathology, First Affiliated Hospital of Kunming Medical University, Kunming, China; ^2^ School of Ecology and Environmental Science, Yunnan University, Kunming, China; ^3^ Department of Cardiovascular Medicine, the Bozhou Hospital Affiliated to Anhui Medical University, Bozhou Anhui, China; ^4^ Department of Neurology, First Affiliated Hospital of Kunming Medical University, Kunming, China; ^5^ Yunnan Province Clinical Research Center for Neurological Diseases, Kunming, China; ^6^ Kunming College of Life Science, University of Chinese Academy of Sciences, Beijing, China

**Keywords:** lncRNA, lung adenocarcinoma, prognosis biomarker, cell proliferation, cell apoptosis, cell growth, cell migration

## Abstract

Long non-coding RNAs (lncRNAs) are tumor-associated biological molecules and have been found to be implicated in the progression of lung adenocarcinoma (LUAD). LncRNA-AP000695.2 (ENSG00000248538) is a long non-coding RNA (lncRNA) that is widely increased in many tumor types including lung adenocarcinoma (LUAD). However, the aberrant expression profile, clinical significance, and biological function of AP000695.2 in human lung adenocarcinoma (LUAD) need to be further investigated. This study mines key prognostic AP000695.2 and elucidates its potential role and molecular mechanism in regulating the proliferation and metastasis of LUAD. Here, we discovered that AP000695.2 was significantly upregulated in lung adenocarcinoma tissues compared with healthy adjacent lung tissue and higher in LUAD cell lines than in normal human bronchial epithelial cell lines. A higher expression of AP000695.2 was positively correlated with aggressive clinicopathological characteristics, and AP000695.2 served as an independent prognostic indicator for the overall survival, disease-free survival, and progression-free survival in patients with LUAD. Receiver operating curve (ROC) analysis revealed the significant diagnostic ability of AP000695.2 (AUC = 0.838). Our *in vivo* data confirmed that AP000695.2 promotes the proliferation, migration, and invasion of LUAD cells. GSEA results suggested that AP000695.2 co-expressed genes were mainly enriched in immune-related biological processes such as JAK-STAT signaling pathway and toll-like receptor signaling pathway. Single-sample GSEA analysis showed that AP000695.2 is correlated with tumor-infiltrating immune cells in lung adenocarcinoma. Our findings confirmed that AP000695.2 was involved in the progression of lung adenocarcinoma, providing a novel prognostic indicator and promising diagnostic biomarker in the future.

## Introduction

Lung cancer is the leading cause of cancer-related death worldwide, according to cancer statistics 2020. The incidence rate of lung cancer ranks second, while the death rate of lung cancer ranks first (Siegel et al., 2020). Lung cancer includes small cell lung carcinoma (SCLC) and non-small cell lung carcinoma (NSCLC). NSCLC includes lung adenocarcinoma (ADC), lung squamous cell carcinoma (SCC), and large-cell lung carcinoma. NSCLC accounts for approximately 85% of all cases (Molina et al., 2008). Despite various treatments applied to the diagnostic and therapeutic for lung cancer, the five-year survival rate of lung cancer remains poor (Reck and Rabe 2017). As an important treatment of lung cancer, chemotherapy is widely used in the clinical treatment of patients. However, the drug resistance of patients to chemotherapeutic drugs has become a major challenge to patient prognosis, leading to lung cancer recurrence and metastasis (Zhang et al., 2020). Cisplatin (DDP) was reported to be a common chemotherapeutic drug which plays an important role in the treatment of lung cancer (Dasari and Bernard Tchounwou 2014). Nevertheless, the drug resistance to chemotherapeutic drugs usually leads to poor treatment effects, leading to malignant progression and recurrence of LUAD (Fennell et al., 2016). Elucidating the complex molecular mechanism underlying drug resistance and identifying the key molecules regulating drug resistance are crucial for the treatment of lung cancer.

LncRNAs are a kind of ncRNAs whose transcripts with a length of more than 200 nucleotides do not have protein-coding potential. Mounting evidence has demonstrated that lncRNA abnormal expression and over-activation are usually involved in cancer initiation and progression (Zhang et al., 2020). LncRNAs can modulate the gene expression via influencing the structure of chromatin (Wang et al., 2011), histone modification (Luco et al., 2010), and to sponging microRNA (Yang et al., 2014). Aberrantly expressed lncRNAs have been reported to correlate with the development and progression of lung cancer (Chen et al., 2020a). It has been confirmed that lncRNA plays an important role in regulating cancer chemoresistance (Zhang et al., 2020). For example, Chen et al. found that lncRNA SNHG14 was upregulated in the A549/DDP cell line compared to that in the A549 cell line, and SNHG14 promotes the DDP-resistance of non-small cell lung cancer cells by modulating the miR-133a/HOXB13 signaling pathway (Xu et al., 2020). Furthermore, it has been confirmed that lincAK126698 improves the expression of *β*-catenin and promotes DDP-induced apoptosis (Yang et al., 2013). Moreover, it has been confirmed that lncRNA bladder cancer-associated transcript 1 was highly expressed in DDP-resistant NSCLC cells and promotes chemoresistance via modulating the miR-17/ATG7 axis (Huang et al., 2019). LncRNA-NNT-AS1 is elevated in DDP-resistant NSCLC tissues and cells, and the overexpression of lncRNA NNT-AS1 boosts cell proliferation and inhibits cell apoptosis by regulating the MAPK signaling pathway (Cai et al., 2018). Wang J. et al (2020) showed that LINC01116 was highly expressed in cisplatin-resistant LUAD specimens and A549/DDP cells. Depletion of LINC01116 reduced cell viability and cell migration, elevated cell apoptosis, and improved the sensitivity to DDP in A549/DDP cells. In our previous study, we developed a new method called CVAA (Cross-Value Association Analysis), which functions without a normalization and distribution assumption. We applied it to large-scale pan-cancer transcriptome data generated by The Cancer Genome Atlas (TCGA) project and successfully discovered numerous new differentially expressed genes (DEGs) (Jiang et al., 2021). AP000695.2 is one of these DEGs. Based on our analysis, AP000695.2 is a long non-coding RNA that is highly expressed in various human cancers, including lung cancer. However, the clinical significance and function of AP000695.2 associated with DDP resistance in LUAD remain to be elucidated.

In this study, we explored the diagnostic and prognostic significance of AP000695.2 in lung adenocarcinoma by data mining in The Cancer Genome Atlas (TCGA) and the Gene Expression Omnibus (GEO) datasets. Subsequently, gene set enrichment analysis (GSEA) was used to examine the possible biological functions and signaling pathways of AP000695.2 in lung adenocarcinoma. Moreover, we also examined the relationship between AP000695.2 expression and immune cell infiltration levels to explore the possible mechanisms by which AP000695.2 affected lung cancer occurrence and progression. Finally, cell viability assay, flow cytometry, colony formation, transwell, and wound healing assays were used to determine the biological function of AP000695.2 in lung adenocarcinoma.

## Materials and Methods

### Data Collection

TCGA-LUAD dataset and clinical information of LUAD patients were downloaded from TCGA website (https://portal.gdc.cancer.gov/repository). AP000695.2 expression data from GSE81089 datasets were downloaded from the GEO database and validated AP000695.2 expression.

### Nomogram Construction and Evaluation

Based on the multivariate Cox analysis results, we established a nomogram to predict the prognosis of LUAD patients. According to the prognosis model, we calculated each patient’s risk score as the total score of each parameter, which could predict the prognosis of LUAD patients. The accuracy estimation of nomogram prediction was obtained from a calibration plot. The nomogram discrimination was determined using a concordance index (C-index), and 1,000 resamples were used in the calculation by the bootstrap approach. In this study, all statistical tests were two-tailed, with a statistical significance level of 0.05.

### Gene Set Enrichment Analysis

Using the clusterProfiler package, the subtype-specific gene expression patterns and potential cellular pathways were elucidated on GSEA software (Subramanian et al., 2005). Based on the AP000695.2 expression level, we divided gene expression data into high-AP000695.2 and low-AP000695.2 groups, and each analysis included 1,000 times of gene set permutations. A *p* value of less than 0.05 was considered statistically significant.

### Immune Infiltration Analysis by ssGSEA

We used a GSVA R package to examine the lung adenocarcinoma immune infiltration of 24 tumor-infiltrating immune cells in tumor samples through ssGSEA (Bindea et al., 2013; Hänzelmann et al., 2013). The correlation between AP000695.2 and infiltration levels of immune cells was analyzed by the Spearman correlation, and these immune cells with the different expression groups of AP000695.2 were analyzed by the Wilcoxon rank-sum test.

### Cell Culture

The BEAS-2B cell line was purchased from the Cell Bank of Kunming Institute of Zoology and cultured in BEGM (Lonza, CC-3170). Lung cancer cell lines, including A549, H1299, and SPC-A1, were purchased from Cobioer, China, with STR documents, and were cultured in an RPMI-1640 medium (Corning) supplemented with 10% fetal bovine serum (FBS) and 1% penicillin/streptomycin.

### Constructs, Lentiviral Preparation, and Establishment of Different Cell Lines

For shRNA knockdown experiments, independent shRNAs targeting a different region of AP000695.2 RNA were constructed using a pLKO.1 vector (Addgene), and the oligo sequences were provided as follows: lentiviruses were generated according to the manufacturer’s protocol, and indicated cells were infected by viruses twice with 48 and 72 h viral supernatants containing 4 μg/ml polybrene, and stable cell lines were established by appropriate puromycin selection. The two independent AP000695.2 targeting sequences are: shRNA#1, 5′-GTG​TTG​TAT​GAC​CCC​GTT​TTC-3' and shRNA#2, 5′-ATA​CGC​ACT​GAC​AAA​CAA​CAC-3'.

### Cell Proliferation Assays

For the cell proliferation assay, a total of 2 × 10^4^ indicated cells were plated into 12-well plates in triplicate, and the exact cell numbers for each day were determined using an automatic cell analyzer countstar. Cell migration assay was performed as previously described (Jiang et al., 2022). To produce a wound, the monolayer cells in a 6-well plate were scraped in a straight line with pipette tips. The plate was then washed with warm PBS to remove detached cells. Photographs of the scratch were taken at indicated time points using a Nikon inverted microscope (Ti-S). The gap width was calculated with GraphPad Prism software. For transwell assay, 1–2×10^4^ cells in 100 μL serum-free medium were plated in an 8.0-μm, 24-well plate chamber insert, with a medium containing 10% FBS at the bottom of the insert. The cells were incubated for 24 h and then fixed with 4% paraformaldehyde for 20 min. After washing, the cells were stained with 0.5% crystal violet-blue. The positively stained cells were examined under the microscope.

### Real-Time RT-PCR Assay

A real-time RT-PCR assay was performed as previously described (Jiang et al., 2022). The primer used in this study is as follows: *β*-actin-F: AAG​TGT​GAC​GTG​GAC​ATC​CGC, *β*-actin-R: CCG​GAC​TCG​TCA​TAC​TCC​TGC​T, AP000695.2-F: GAT​GAA​AGA​CCG​CGT​TGT​TT, and AP000695.2-R: CTCTTCGGAGGAAATAC.

### Statistical Analysis

Data are represented as mean ± SEM, and error bars indicate SEM. *p* values were calculated by either unpaired or paired two-tailed Student’s *t*-test, *p < 0.05, **p < 0.01, and ***p < 0.001. All analyses were performed using GraphPad Prism software (GraphPad Software, Inc.).

## Results

### The Expression and Prognostic Value of AP000695.2 in Pan-Cancer

**FIGURE 1:**
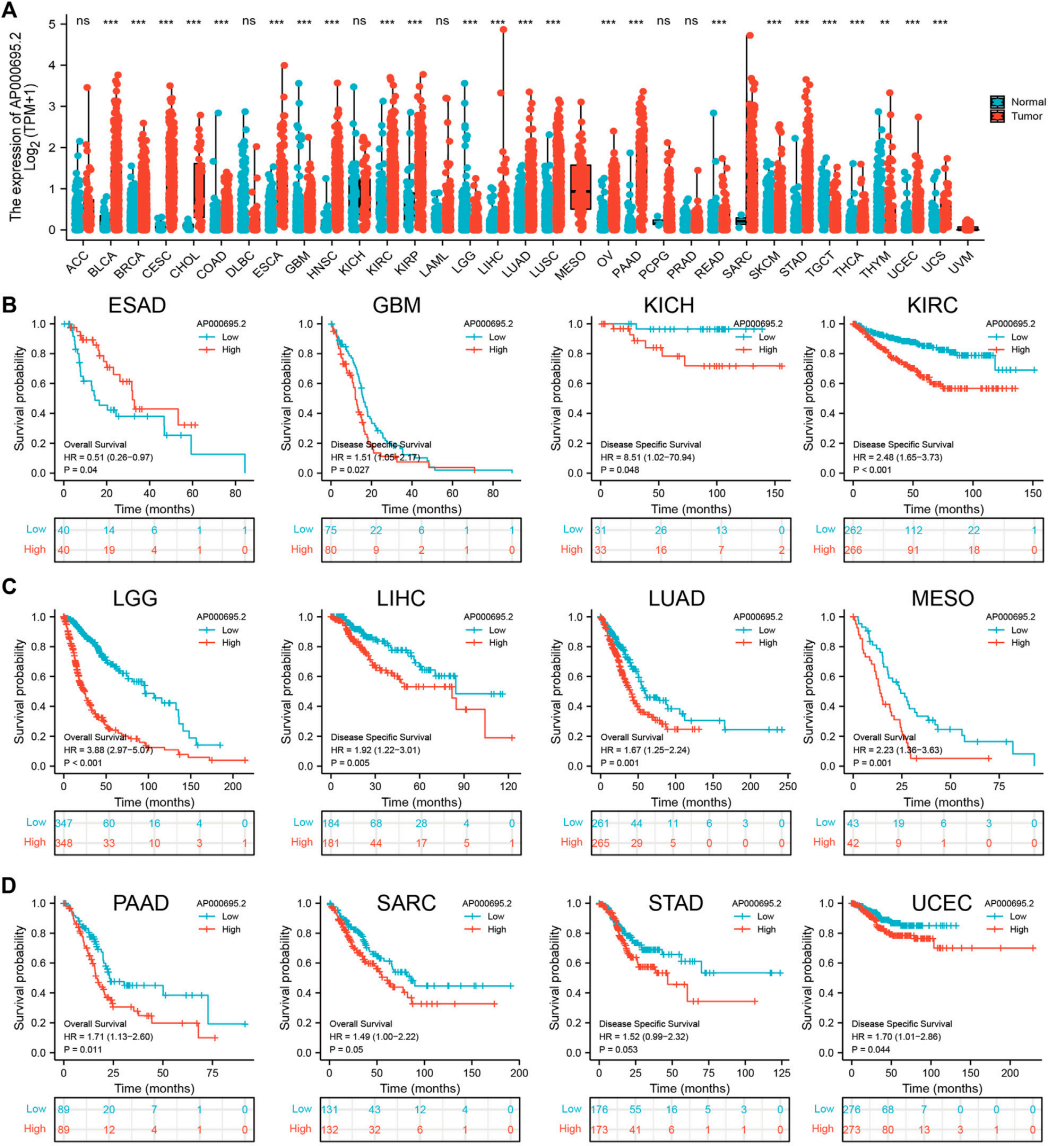
Expression level and prognosis of lncRNA-AP000695.2 in human cancer. **(A)** Expression of lncRNA-AP000695.2 in normal and tumor tissues in TCGA and GTEx data, based on the best cutoff score to distinguish high- and low-expression groups. **(B–D)** Correlation between AP000695.2 expression and cancer survival prognosis. *p < 0.05, **p < 0.01, and ***p < 0.001.

To explore the expression of AP000695.2 in diverse human cancer, the AP000695.2 RNA expression data of multiple human cancers and normal tissues were examined. Based on the best cutoff score, we found that AP000695.2 expression was higher in various tumor tissues. As shown in Figure 1A, AP000695.2 expression was extremely significant in bladder urothelial carcinoma (BLCA), breast invasive carcinoma (BRCA), cervical squamous cell carcinoma (CESC), cholangiocarcinoma (CHOL), colon adenocarcinoma (COAD), esophageal carcinoma (ESCA), glioblastoma multiforme (GBM), head and neck squamous cell carcinoma (HNSC), kidney renal clear cell carcinoma (KIRC), kidney renal papillary cell carcinoma (KIRP), liver hepatocellular carcinoma (LIHC), lung adenocarcinoma (LUAD), lung squamous cell carcinoma (LUSC), ovarian serous cystadenocarcinoma (OV), pancreatic adenocarcinoma (PAAD), skin cutaneous melanoma (SKCM), stomach adenocarcinoma (STAD), uterine corpus endometrial carcinoma (UCEC), and uterine carcinosarcoma (UCS) (Figure 1A). Furthermore, we found that AP000695.2 with high expression was associated with adverse clinical outcomes in ESAD, GBM, KICH, KIRC, LGG, LIHC, LUAD, MESO, PAAD, SARC, STAD, and UCEC (Figures 1B–D).

### The Correlation Between AP000695.2 Expression Levels and Clinical Parameters in Lung Adenocarcinoma

**FIGURE 2:**
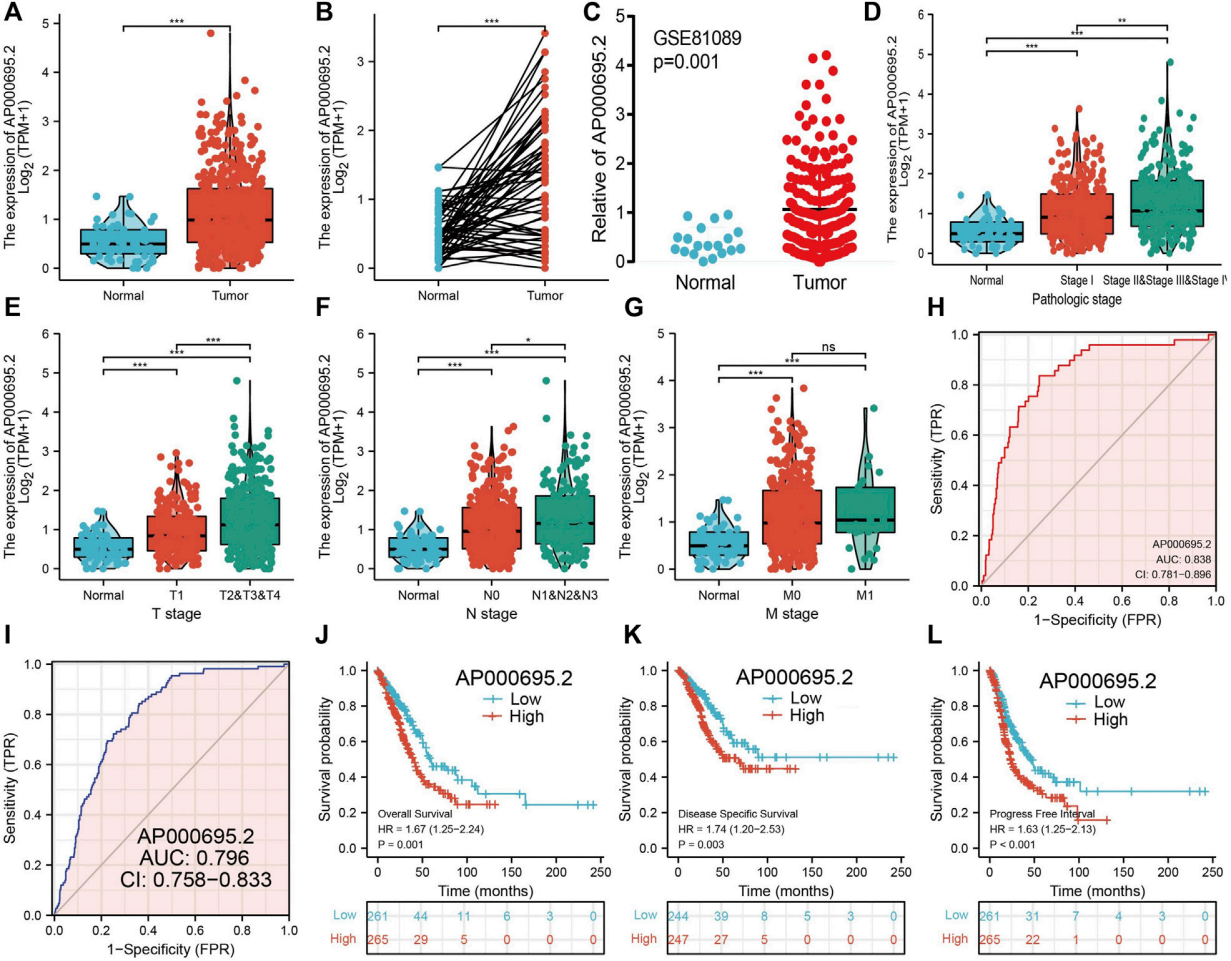
Clinical significance of lncRNA-AP000695.2 in lung adenocarcinoma. **(A)** Expression of lncRNA-AP000695.2 in lung cancer based on TCGA dataset. **(B)** Expression levels of lncRNA-AP000695.2 in 59 paired adjacent normal tissues and paired samples. **(C)** Expression levels of lncRNA-AP000695.2 in lung cancer based on the GEO dataset. **(D–G)** Correlation between lncRNA-AP000695.2 expression and clinical parameters includes pathological and TNM stages. **(H–I)** ROC curves were used to determine the diagnostic value of lncRNA-AP000695.2 in lung adenocarcinoma based on TCGA-LUAD and GEO datasets. **(J–L)** Kaplan–Meier survival curves showed that lung adenocarcinoma patients with high-lncRNA-AP000695.2 expression exhibited poor overall survival, disease-specific survival, and progression-free survival based on TCGA-LUAD dataset. *p < 0.05, **p < 0.01, and ***p < 0.001.

We simultaneously analyzed the expression profiles of AP000695.2 (ENSG00000248538) based on TCGA database. Results confirmed that AP000695.2 was highly expressed in lung adenocarcinoma compared to the normal samples (Figure 2A). There were 59 pairs of lung adenocarcinoma cancer samples and matched adjacent normal samples from TCGA data. We found that AP000695.2 was elevated in lung adenocarcinoma samples than in matched adjacent normal samples (Figure 2B). Moreover, we found that AP000695.2 was increased in lung cancer tissues by analyzing the GEO dataset (Figure 2C). To examine the clinical relevance of AP000695.2 in LUAD, 535 LUAD patients with clinical characteristics were divided into two subgroups based on the mean value of relative AP000695.2 expression. We then explored the correlations between AP000695.2 expression and clinical features, including pathologic stage, TNM stage, and residual tumor. We found that AP000695.2 expression was significantly correlated with pathologic stage, TNM stage, and residual tumor (Figures 2D–G). Furthermore, ROC analysis showed that AP000695.2 could be used to differentiate LUAD patients from normal controls with a specificity (AUC = 0.838) (Figure 2H). This result is also validated by the GEO dataset (Figure 2I). Moreover, Kaplan–Meier analysis showed that LUAD patients with higher AP000695.2 expression were associated with adverse overall survival (OS), disease-free survival, and progression-free survival (PFS) (Figures 2J-L).

### Univariate and Multivariate Cox Regression Analyses

Univariate Cox regression analysis was conducted to determine whether the AP000695.2 expression level and pathologic stage might be valuable prognostic biomarkers in TCGA-LUAD cohort. In univariate Cox regression analysis, the high expression of AP000695.2, pathologic stage, and TNM stage was associated with overall survival in LUAD patients. To assess whether AP000695.2 could be an independent prognostic factor for LUAD patients, multivariate Cox survival analysis showed that AP000695.2 expression could be an independent prognostic factor for LUAD (Tables 1).

**TABLE 1:** Univariate and multivariate Cox regression analyses of different parameters on overall survival in lung adenocarcinoma.

Characteristic	Total (N)	Univariate analysis		Multivariate analysis	
		Hazard ratio (95% CI)	*p*-value	Hazard ratio (95% CI)	*p*-value
T stage	523				
T1 and T2	457				
T3 and T4	66	2.317 (1.591–3.375)	<0.001	1.638 (1.018–2.635)	0.042
N stage	510				
N0 and N1	437				
N3 and N2	73	2.321 (1.631–3.303)	<0.001	1.293 (0.626–2.674)	0.488
Pathologic stage	518				
Stage II and Stage I	411				
Stage IV and Stage III	107	2.664 (1.960–3.621)	<0.001	1.802 (0.839–3.871)	0.131
M stage	377				
M0	352				
M1	25	2.136 (1.248–3.653)	0.006	1.192 (0.541–2.626)	0.664
AC022784 1	526	1.251 (1.141–1.372)	<0.001	1.168 (1.053–1.296)	0.003

**FIGURE 3:**
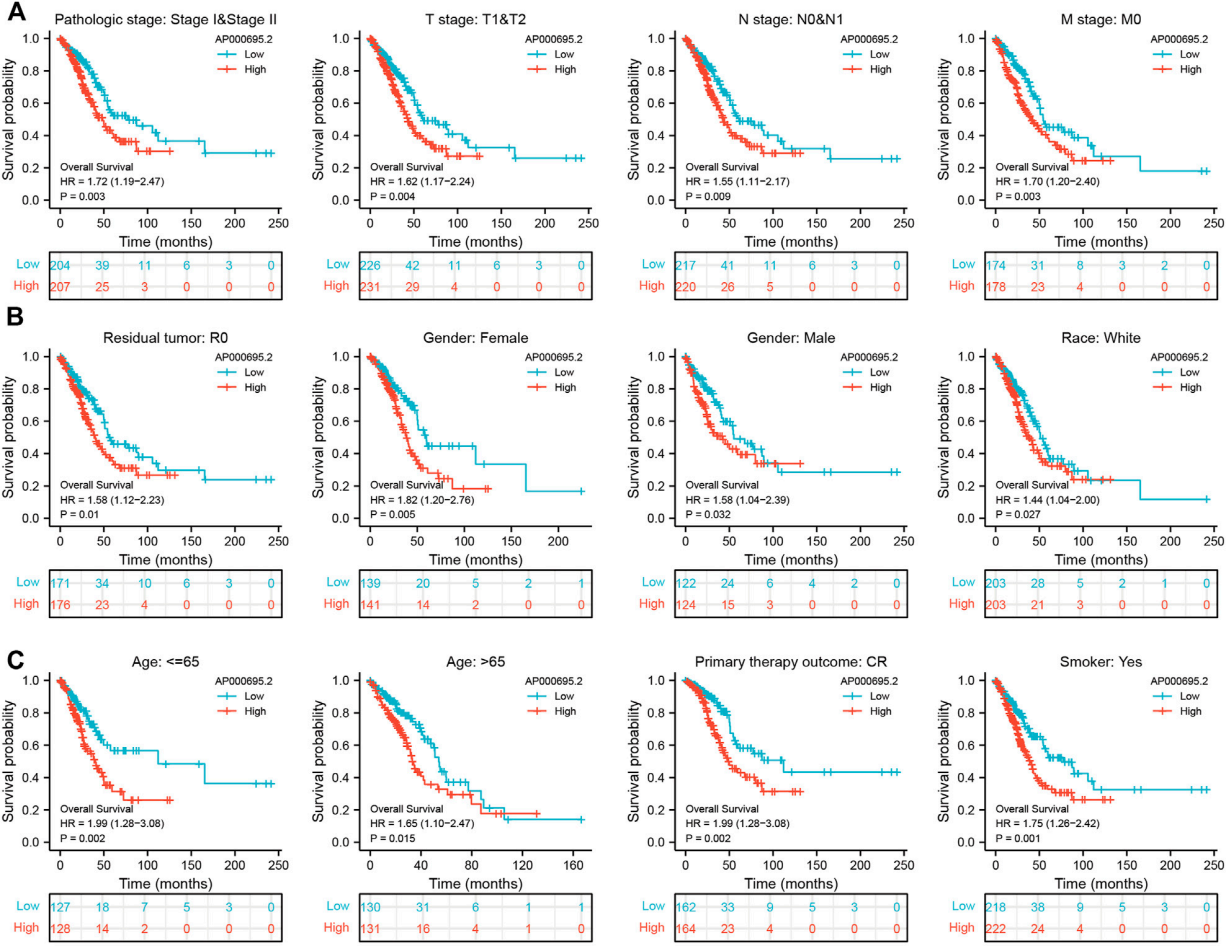
Kaplan–Meier survival analyses for prognostic values of AP000695.2 in different subgroups stratified by clinical features. **(A–C)** Kaplan–Meier curves for overall survival of AP000695.2 in subgroups including stages I-II, T1-T2, N0-N1, M0, R0, female, male, race, white, age>65years, age <65years, CR, and smoker in TCGA-LUAD cohort.

To examine whether the prognostic values of AP000695.2 was applicable to other clinical features, all patients were divided into various subgroups based on the clinical features. Survival analyses performed in the subgroups indicated that AP000695.2 performed well in subgroups such as stages I-II (*p* = 0.003), T1-T2 (*p* = 0.004), N0-N1 (*p* = 0.009), M0 (*p* = 0.003), R0 (*p* = 0.01), female (*p* = 0.005), male (*p* = 0.032), race, white (*p* = 0.027), >65years (*p* = 0.015), <65years (*p* = 0.002), CR (*p* = 0.002), and smoker (*p* = 0.001) in TCGA-LUAD cohort (Figures 3A–C).

### Validation of the Prognostic Significance of AP000695.2 in Lung Adenocarcinoma Cohorts

**FIGURE 4:**
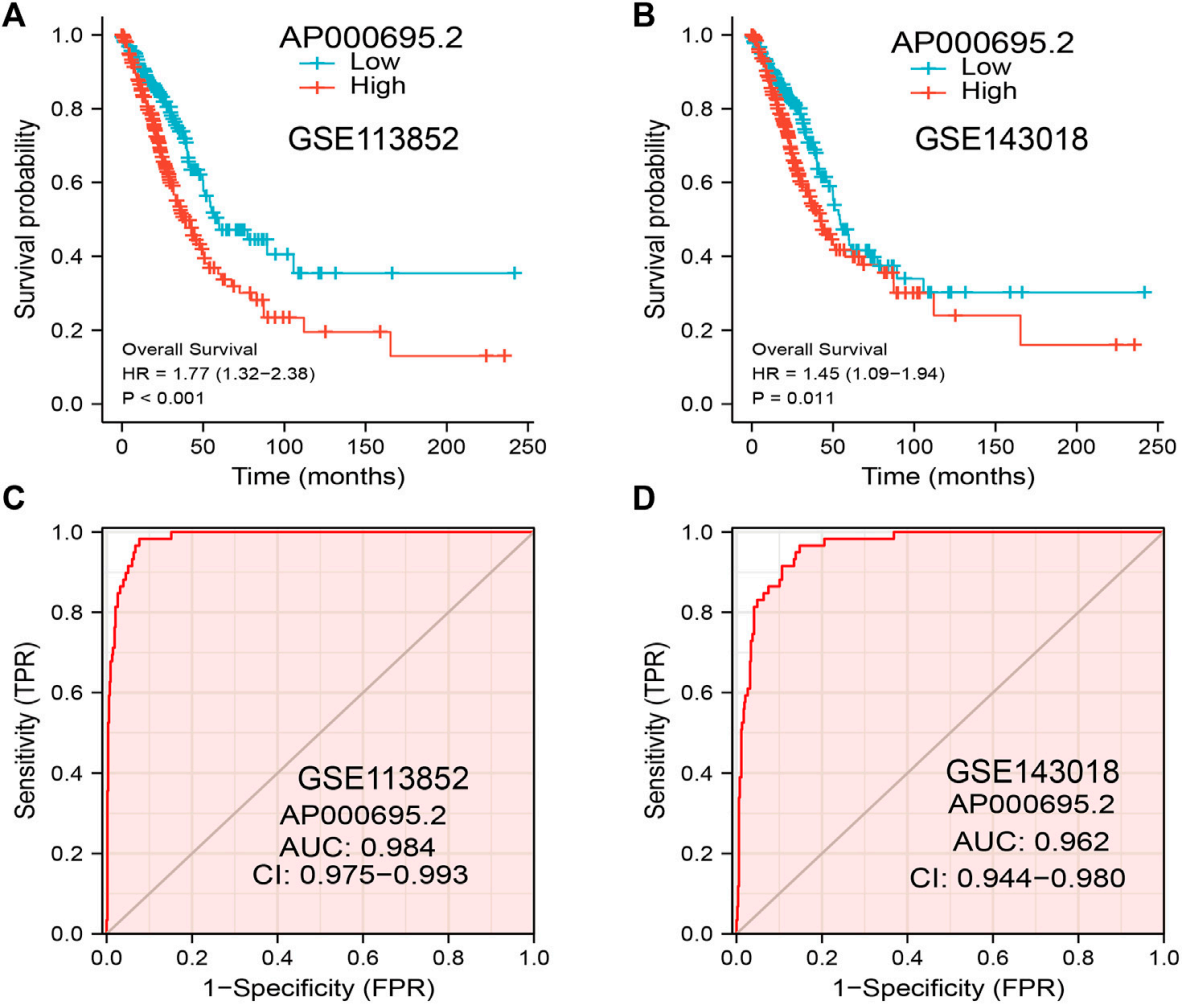
Predictive powers for the prognosis of AP000695.2 in LUAD patients. **(A–B)** Kaplan–Meier survival validation of the prognostic values of AP000695.2 in LUAD patients based on the GEO cohorts. **(C–D)** ROC curves show the AUC of AP000695.2 for predicting the overall survival of LUAD patients.

The GEO dataset was used to validate the prognostic significance of AP000695.2 in LUAD, and we showed that the LUAD patients in the high-expression group had shorter OS in the GEO cohorts (Figures 4A,B). Moreover, the ROC curve was also used to assess the predictive power of AP000695.2 in OS, and the AUC for the overall survival rate of LUAD patients was 0.984 and 0.962 in the GEO cohort (Figures 4C,D). Taken together, these results suggest that AP000695.2 is a moderately sensitive index for predicting the prognosis of LUAD patients and can act as an effective prognostic biomarker in LUAD.

### Construction and Validation of an AP000695.2-Based Nomogram

**FIGURE 5:**
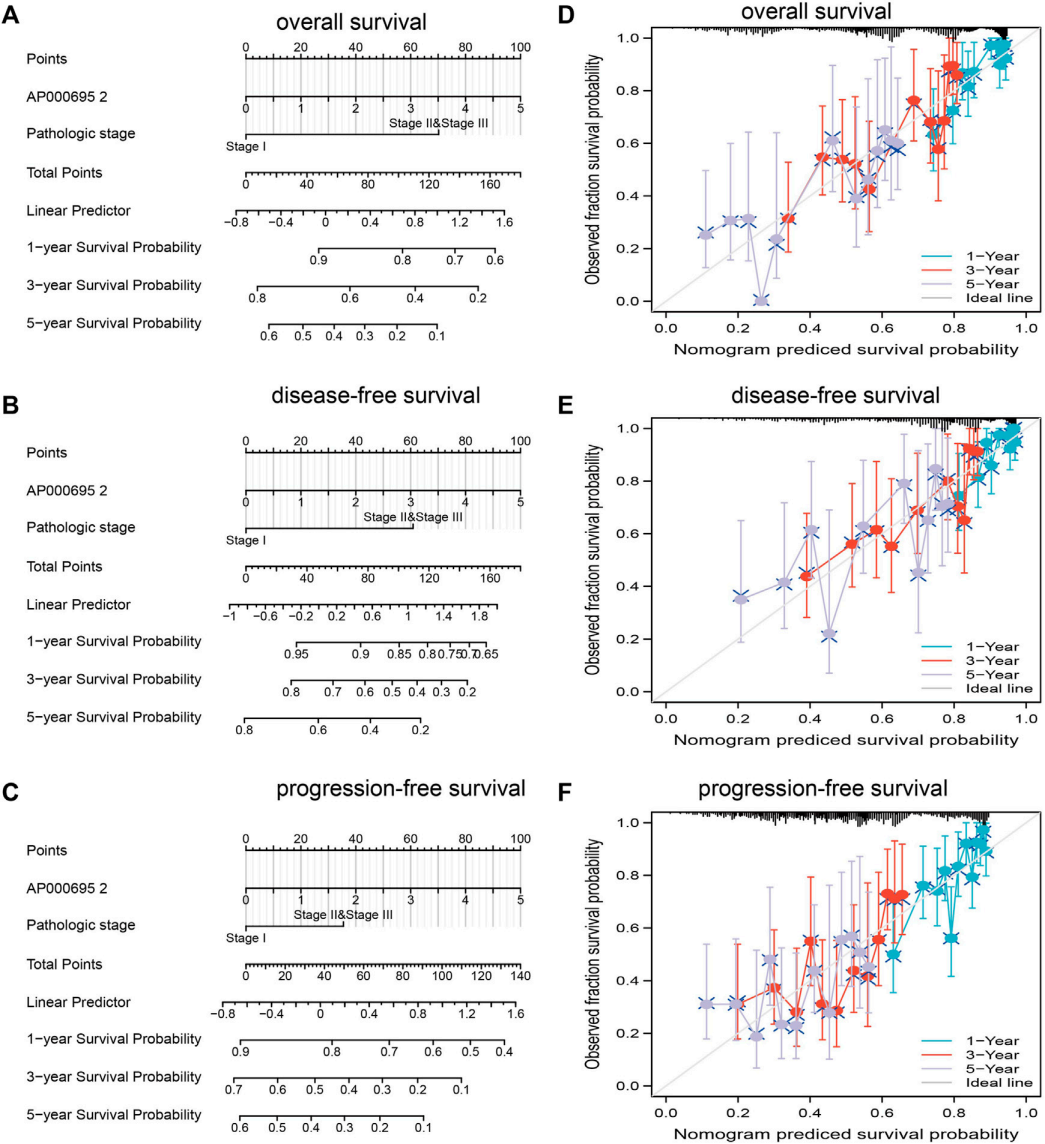
Construction and performance validation of the lncRNA-AP000695.2-based nomogram for lung adenocarcinoma patients. **(A–D)** Nomogram to predict the overall survival, disease-free survival, and progression-free survival of lung cancer patients, and the calibration curve and Hosmer–Lemeshow test of nomograms in TCGA-lung adenocarcinoma cohort for overall survival, disease-specific survival, and progression-free survival.

The multivariate analysis result confirmed that AP000695.2 is an independent prognostic factor in LUAD. We then constructed a prediction model for overall survival, disease-free survival, and progression-free survival by integrating AP000695.2 expression and pathologic stage. We established a nomogram to integrate AP000695.2 as an LUAD biomarker, and higher total points on the nomogram for OS, PFS, and DFS indicated a worse prognosis (Figures 5A–F). These findings indicated that the nomogram could well predict clinical outcomes of lung adenocarcinoma patients.

### AP000695.2-Related Signaling Pathways Based on Gene Set Enrichment Analysis

**FIGURE 6:**
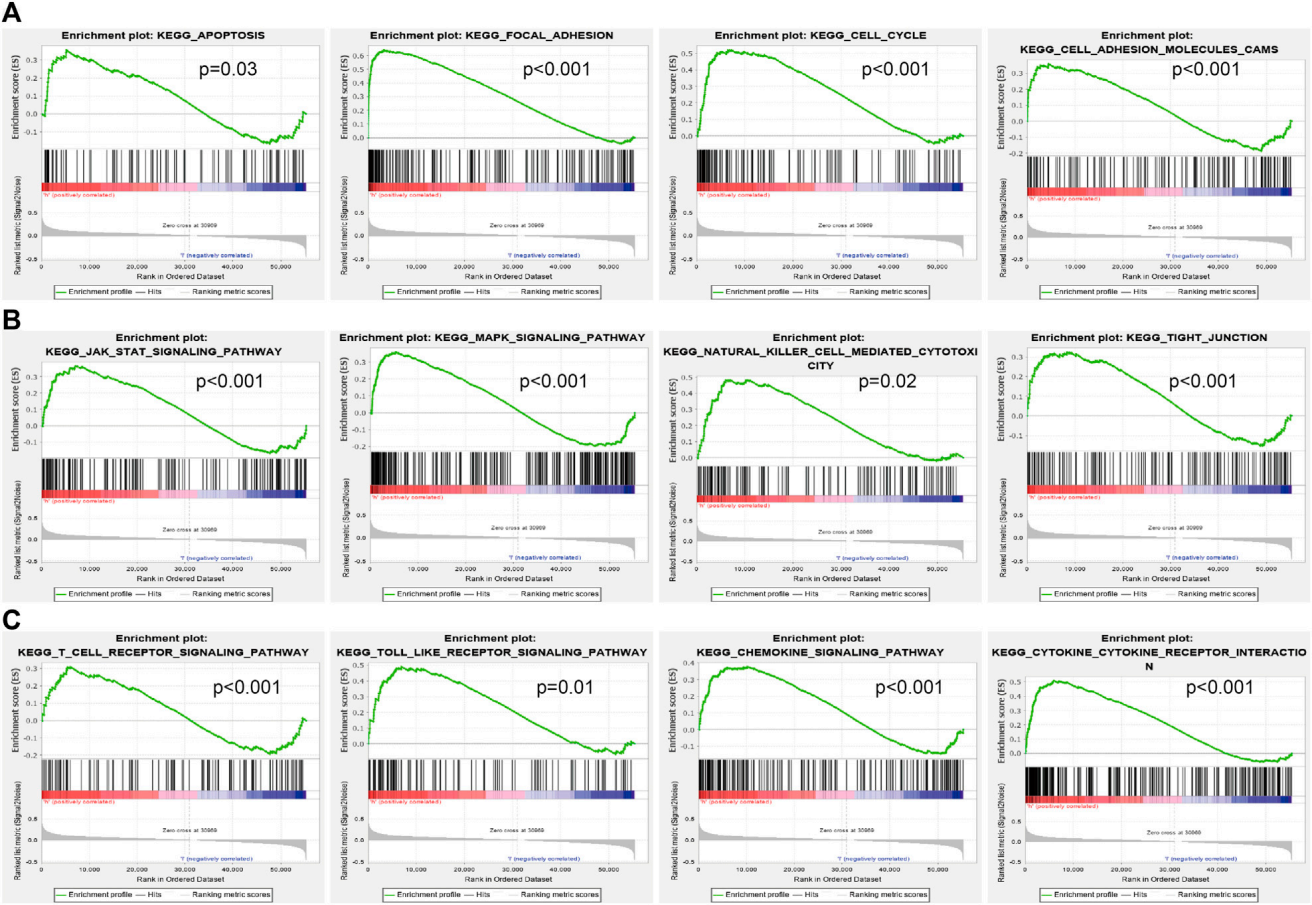
Identification of lncRNA-AP000695.2-related signaling pathways in lung adenocarcinoma. **(A–B)** Top 12 significant KEGG pathways associated with AP000695.2 were examined by GSEA software.

To explore the influence of potential signaling pathways of AP000695.2 in LUAD, gene set enrichment analysis (GSEA) was performed on the datasets with high and low expressions of AP000695.2. We only selected the top nine datasets with a high normalized enrichment score (NES) and significant *p*-value. The results showed that cell apoptosis, focal adhesion, cell cycle, cell adhesion molecules cams, JAK-STAT signaling pathway, MAPK signaling pathway, natural killer cell-mediated cytotoxic, tight junction, T-cell receptor signaling pathway, toll-like receptor signaling pathway, chemokine signaling pathway, and cytokine–cytokine receptor interaction were significantly enriched in the high-AP000695.2 expression group in TCGA datasets (Figures 6A–C). These results suggested that AP000695.2 might participate in the regulation of cell apoptosis and immune response in LUAD.

### Correlation Between AP000695.2 Expression and Immune Infiltration

**FIGURE 7:**
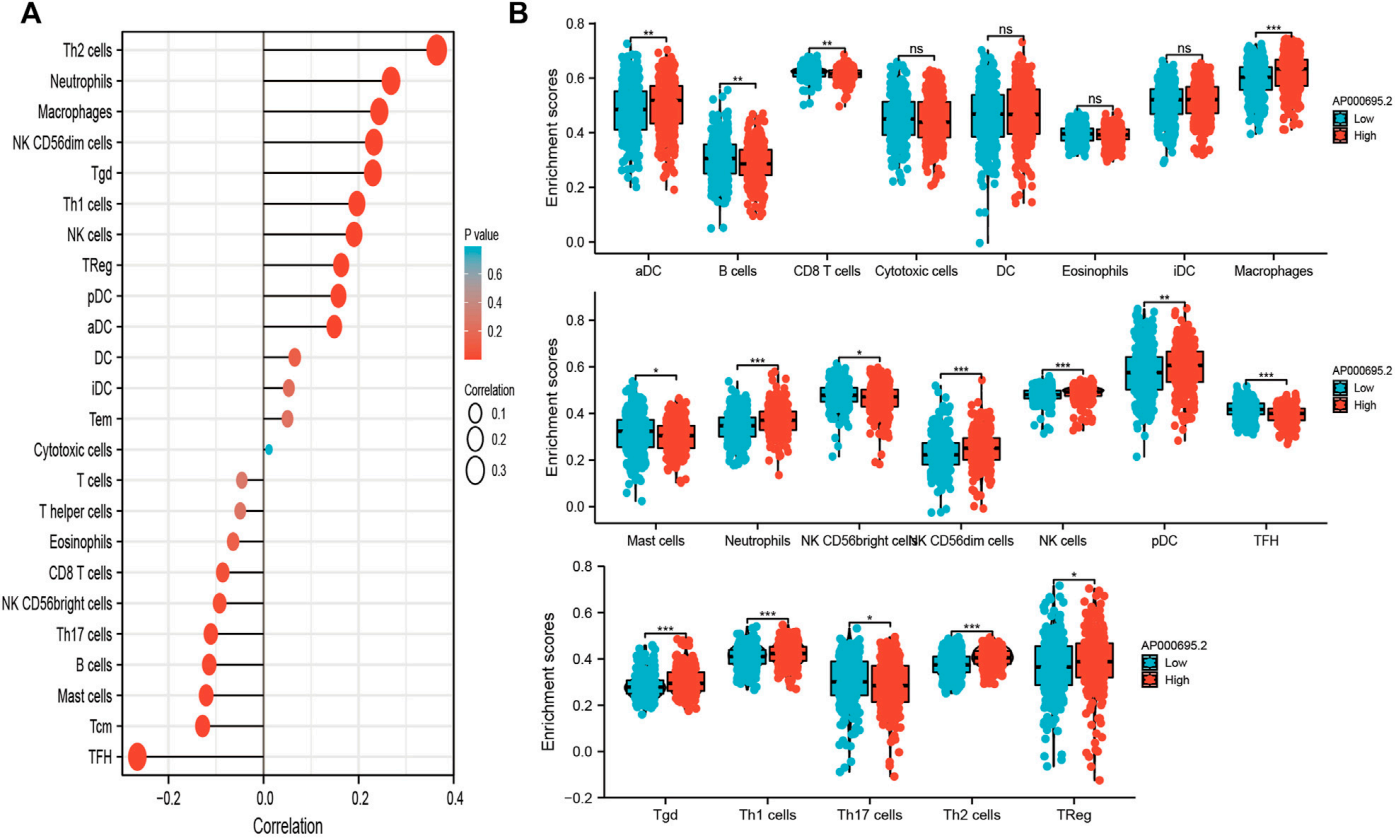
Correlation analysis of lncRNA-AP000695.2 expression and infiltration levels of immune cells in LUAD tissues. **(A)** Correlation between the relative abundances of 24 immune cells and lncRNA AP000695.2 expression level. **(B)** Box plots of the correlations between lncRNA-AP000695.2 or molecular model expression and infiltration levels of immune cells. *p < 0.05, **p < 0.01, and ***p < 0.001.

ssGSEA with Spearman’s rank correlation was utilized to determine the relationship between AP000695.2 expression and infiltrating immune cells in LUAD. The results confirmed that AP000695.2 was positively correlated with infiltration levels of Th2 cells, neutrophils, macrophages, NK CD56dim cells, Ted, Th1 cells, NK cells, TReg, pDC, and aDC (*p* < 0.001) and was negatively correlated with that of eosinophils, CD8 T cells, NK CD56bright cells, Th17 cells, B cells, mast cells, Tcm, and TFH (Figure 7A). Furthermore, we found that patients in the AP000695.2 high-expression group showed an increase in the numbers of infiltrating aDC, macrophages, neutrophils, NK CD56dim cells, NK cells, pDC, Tgd, Th1 cells, and Th2 cells. On the contrary, patients in the AP000695.2 low-expression group showed a reduction in the numbers of infiltrating eosinophils, CD8 T cells, NK CD56bright cells, Th17 cells, B cells, mast cells, Tcm, and TFH (Figure 7B).

### AP000695.2 Promotes Proliferation and Migration of Lung Adenocarcinoma Cells *In Vitro*

**FIGURE 8:**
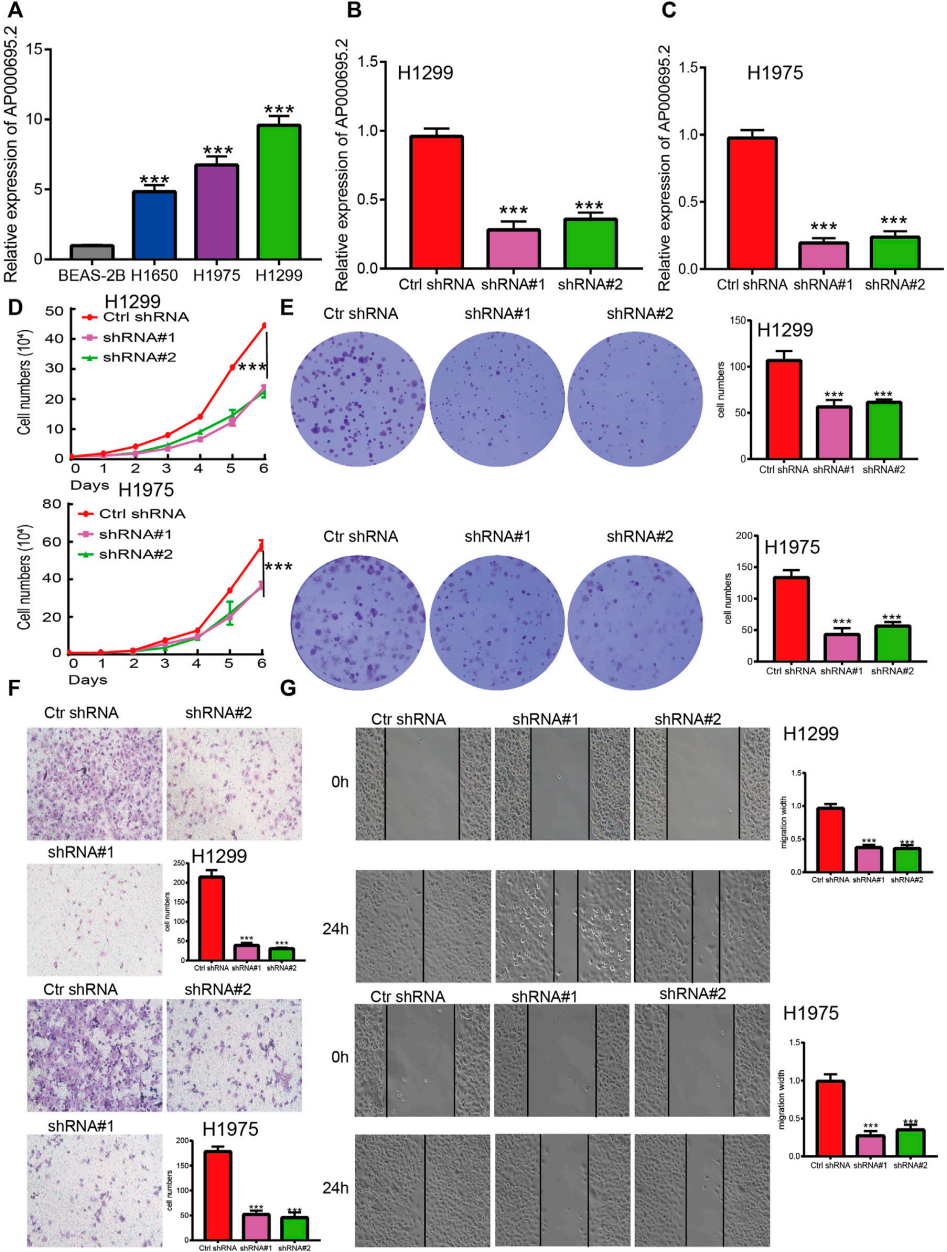
lncRNA-AP000695.2 promotes LUAD cell proliferation, migration, and invasion *in vitro*. **(A)** Relative expression level of lncRNA AP000695.2 in lung adenocarcinoma cancerous cell lines, including H1650, H1975, and H1299 examined by real-time RT-PCR, compared with normal human bronchial epithelial cell lines: BEAS-2B. **(B–C)** Establishment of lncRNA-AP000695.2 knockdown cell lines in H1299 and H1975 cells verified by real-time RT-PCR. **(D–E)** Knockdown of lncRNA-AP000695.2 significantly inhibits cell proliferation in H1299 and H1975 cells as measured by BrdU and colony formation. **(F–G)** Knockdown of lncRNA AP000695.2 dramatically inhibits H1299 and H1975 cells’ migration ability examined by transwell and wound healing assays. *p < 0.05, **p < 0.01, and ***p < 0.001.

The aforementioned studies suggested that AP000695.2 expression was elevated in LUAD tissues, and AP000695.2 may involve in the progression of LUAD. To further determine the function of AP000695.2 in LUAD, we found that AP000695.2 was elevated in H1650, H1975, and H1299 lung cancer cell lines compared to the human bronchial epithelial cells (BEAS-2B) (Figure 8A). Furthermore, specific shRNA for AP000695.2 was utilized to construct H1299 and H1975 cells with stable knockdown of AP000695.2 expression. The knockdown efficiencies were examined by qRT-PCR assay (Figures 8B,C). The knockdown of AP000695.2 inhibited the cell proliferation capacity of H1299 and H1975 cells (Figures 8D,E) on BrdU and colony assays. Moreover, transwell assay and wound healing showed that depletion of the AP000695.2 expression level significantly reduced the cell migration and invasion abilities of H1299 and H1975 cells (Figures 8F,G). These results confirmed that AP000695.2 plays a role of an oncogene in lung cancer cells.

### AP000695.2 Facilitates Cisplatin Resistance in Lung Adenocarcinoma *In Vitro*

**FIGURE 9:**
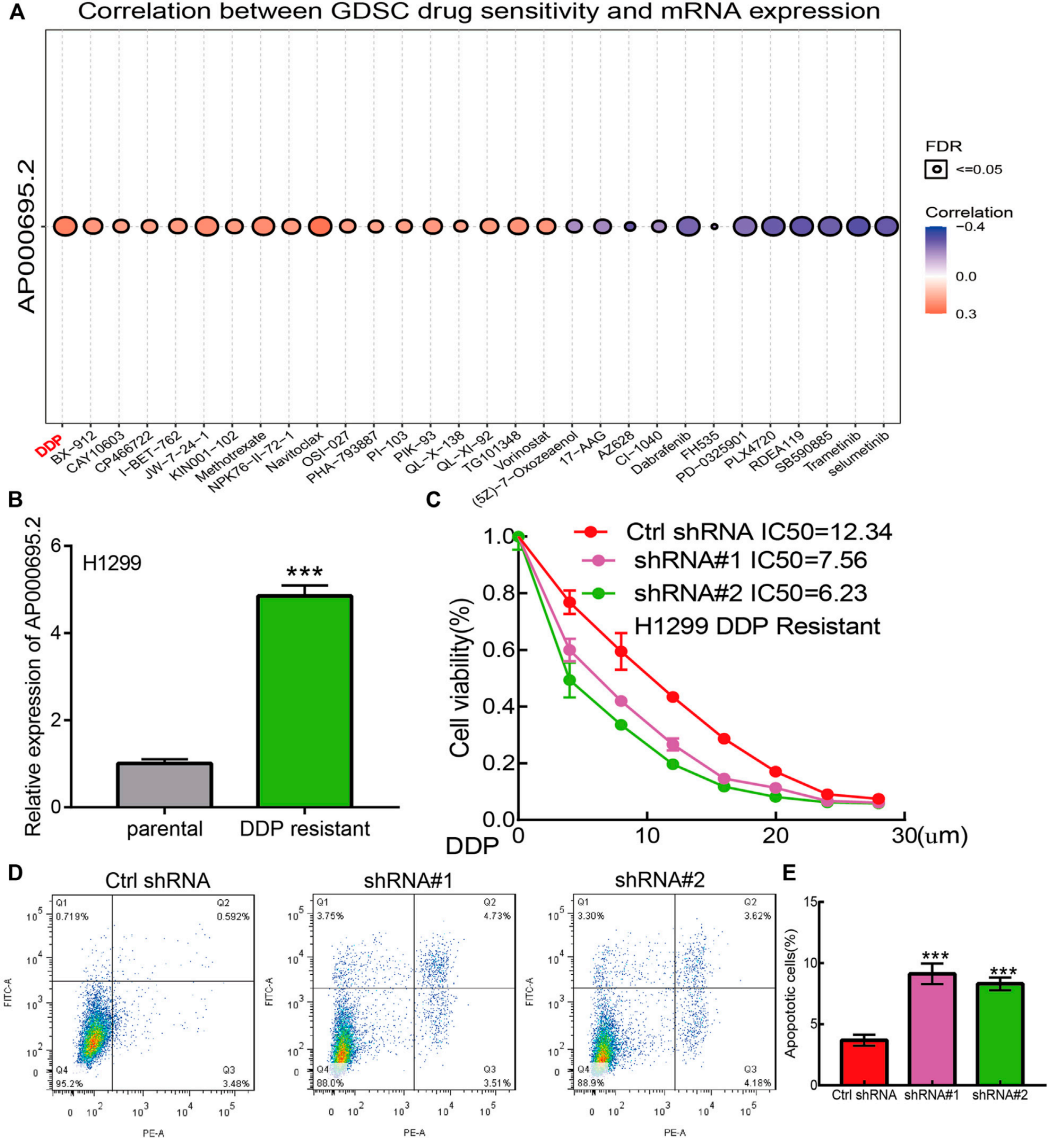
lncRNA-AP000695.2 contributes to DDP resistance of LUAD. **(A)** Relationships between AP000695.2 expression and different drugs were examined by the GDSC database. **(B)** Expression of AP000695.2 in DDP-resistant and matched parental LUAD cells, normalized to 18sRNA expression. **(C)** AP000695.2 knockdown increases the sensitivity of H1299/CDDP-resistant cells to CDDP, detected by CCK-8 assay. **(D–E)** AP000695.2 knockdown increases the CDDP-induced apoptosis rate in H1299/DDP-resistant cells detected by FCM.

We utilized the GDSC database to explore the potential drug that correlated with AP000695.2. The results confirmed that AP000695.2 was positively correlated with the diverse drug, including cisplatin (DDP) (r = 0.43, *p* < 0.001) which was reported to be a common chemotherapeutic drug for LUAD patients (Figure 9A). To further determine the function of DDP resistance in LUAD, we utilized DDP-sensitive, parental LUAD cell lines H1299 and their isogenic DDP-resistant counterparts H1299 as experimental cell lines. We found that AP000695.2 was elevated in H1299-DDP-resistant cells than in H1299 DDP-sensitive cells (Figure 9B). Then, we treated LUAD cells with DDP and calculated the IC_50_ value. The results confirmed that AP000695.2 knockdown significantly decreased the IC_50_ value of DDP (Figure 9C). Likewise, AP000695.2 knockdown significantly promotes the DDP-induced cell apoptosis in H1299-DDP-resistant cells (Figures 9D,E). Overall, an increased AP000695.2 expression could promote DDP resistance of LUAD cells.

## Discussion

Despite there being many different treatments, the prognosis of lung cancer is an advanced stage and the survival rate of patients is very low (Hirsch et al., 2017). The initiation and progression of lung cancer is an exceedingly complicated process that involves genetic mutations, tumor microenvironment, and the dysregulation of epigenetic pathways (Govindan et al., 2012; Duruisseaux and Esteller 2018). It has been shown that lncRNAs maybe serve as effective and specific molecular markers for lung cancer diagnosis. For example, NEAT1 was highly expressed in NSCLC tissues, and its higher expression was associated with the TNM stage and lymphatic metastasis, and the ROC curve of NEAT1 in NSCLC was 0.878, which showed good diagnostic potential in NSCLC (Jen et al., 2017). Li et al. reported that lncRNA UPLA1 was overexpressed in NSCLC tissues and correlated with tumor size and lymph node metastasis, and lncRNA UPLA1 had an ROC curve of 0.756 for discriminating NSCLC from normal controls, with a sensitivity and specificity of 76.2% and 72.1%, respectively (Han et al., 2020). In this study, we found that lncRNA-AP000695.2 was highly expressed in LUAD and correlated with adverse clinical features, including pathologic stage, TNM stage, and residual tumor. The survival analysis results showed that patients with high expression of AP000695.2 correlated with OS, DFS, and PFS in the LUAD patients of TCGA data.

Furthermore, univariate and multivariate analyses as standard and reliable statistical methods were utilized to confirm whether lncRNA can be regarded as an independent tumor marker for predicting the prognosis of lung cancer patients. By univariate and multivariate analyses, some lncRNAs have also been identified as independent prognostic markers in lung cancer. For instance, lncRNA CASC9 was shown to be increased in NSCLC and correlated with poor prognosis. Forced lncRNA CASC9 expression promotes NSCLC cell proliferation and chemoresistance via epigenetic repression of DUSP1, and univariate and multivariate analyses reported that lncRNA CASC9 can be regarded as independent prognostic markers in lung cancer (Chen et al., 2020b). In this study, we confirmed that AP000695.2 expression could be an independent prognostic factor for LUAD. We also established a nomogram to integrate AP000695.2 as an LUAD biomarker, and higher total points on the nomogram for overall survival, progression-free survival (PFS), and disease-specific survival (DSS) indicated a worse prognosis.

LncRNAs are a class of RNAs with more than 200 nucleotides in length. Although lncRNAs do not have protein-coding capabilities, they play essential roles in various biological processes and diseases (Wang et al., 2018). The aberrant regulation of lncRNAs is associated with tumorigenesis, metastasis, and drug resistance (Liang et al., 2021). It has been confirmed that lncRNA TUC338 via activating the MAPK pathway leads to lung cancer progression (Zhang et al., 2018). Additionally, You et al. found that lncRNA-RP11-468E2.5 regulates colorectal cancer cell proliferation and promotes apoptosis by modulating the JAK/STAT signaling pathway by targeting STAT5 and STAT6 (Jiang et al., 2019). For example, Li et al. found that ZFPM2-AS1 was found to facilitate cell proliferation, migration, and invasion via involvement in the JAK-STAT and AKT pathways in NSCLC (Wang X et al., 2020). Moreover, Guo et al. found that lncRNA- XLOC_098,131, via sponging miR-548s and thereby upregulation of FOS expression, leads to the production of more immunoglobulins and the promotion of antigen presentation (Fan et al., 2019). In this study, we found that the high expression of AP000695.2 was highly associated with cell apoptosis, focal adhesion, cell cycle, cell adhesion molecules cams, JAK-STAT signaling pathway, MAPK signaling pathway, natural killer cell-mediated cytotoxic, tight junction, T-cell receptor signaling pathway, toll-like receptor signaling pathway, chemokine signaling pathway, and cytokine–cytokine receptor interaction that were significantly enriched, which indicated that AP000695.2 might have a crucial role in immune response regulation and cell proliferation. The JAK-STAT signaling pathway was reported to play crucial roles in the regulation of intracellular signal transduction and cellular immune response. These results indicated that AP000695.2 may be correlated with the JAK-STAT signaling pathway, MAPK signaling pathway, and toll-like receptor signaling pathway in cancer development and progression.

The tumor microenvironment plays a crucial role in cancer progression and can be used as a biomarker for diagnosis and prognosis (Lee and Cheah 2019). For instance, tumor-associated macrophages are regarded as essential components of the tumor microenvironment and play critical roles in the modulation of cancer progression (Pan et al., 2020). In this finding, we found that AP000695.2 expression was positively associated with infiltration levels of Th2 cells, neutrophils, macrophages, NK CD56dim cells, Tgd, Th1 cells, NK cells, TReg, pDC, and aDC (*p* < 0.001) and was negatively correlated with that of eosinophils, CD8 T cells, NK CD56bright cells, Th17 cells, B cells, mast cells, Tcm, and TFH. Furthermore, we found that patients in the AP000695.2 high-expression group showed an increase in the numbers of infiltrating aDC, macrophages, neutrophils, NK CD56dim cells, NK cells, pDC, Tgd, Th1 cells, and Th2 cells. This correlation means that AP000695.2 expression was significantly positively correlated with the infiltrating levels of aDC, macrophages, neutrophils, NK CD56dim cells, NK cells, pDC, Tgd, Th1 cells, and Th2 cells in LUAD. On the contrary, patients in the AP000695.2 low-expression group showed a reduction in the numbers of infiltrating eosinophils, CD8 T cells, NK CD56bright cells, Th17 cells, B cells, mast cells, Tcm, and TFH. This correlation means that AP000695.2 expression was significantly negatively correlated with the infiltrating levels of eosinophils, CD8 T cells, NK CD56bright cells, Th17 cells, B cells, mast cells, Tcm, and TFH in LUAD. Based on the aforementioned findings, we proposed that AP000695.2 may be involved in the immune response by affecting diverse immune cells infiltrating in the tumor microenvironment of lung cancer. In *in vitro* assay, we show that knockdown of AP000695.2 in H1299 and H1975 inhibited cell proliferation and migration.

Cisplatin is a common drug in lung cancer chemotherapy. However, owing to primary or acquired drug resistance, DDP has not achieved a satisfactory therapeutic effect in most LUAD patients. Therefore, identifying and validating the key regulators involved in drug resistance, especially epigenetic modifications, can provide crucial information for overcoming DDP resistance in LUAD. In this finding, by the analysis of TCGA and GDSC datasets, we found the key lncRNAs involved in DDP resistance in LUAD. Among these lncRNAs, AP000695.2 was elevated in DDP-resistant LUAD cells than in DDP-sensitive cells, confirming that AP000695.2 was related to DDP resistance. Then, we found that the knockdown of AP000695.2 significantly improved the sensitivity of lung cancer cells to DDP and induced cell apoptosis. Through loss-of-function experiments performed *in vitro*, we uncover that AP000695.2 promoted DDP resistance in LUAD. To our knowledge, this finding is the first to confirm the crucial role of AP000695.2 in DDP resistance in LUAD.

This study improves our understanding of the correlation between AP000695.2 and LUAD, but some limitations still exist. First, although we explored the correlation between AP000695.2 and immune infiltration in LUAD patients, there is a lack of experiments to validate the function of AP000695.2 in the tumor microenvironment regulation of LUAD. Second, we uncover that depletion of AP000695.2 inhibits cell proliferation and cell migration of LUAD cells. However, the potential molecular mechanisms of AP000695.2 in cancer progression need to be explored in further studies. Third, we did not conduct the Western blot experiments to detect the expression of the related signaling pathway molecules (JAK/MAPK, etc.) after knocking down AP000695.2 in lung cancer cells. In the future, we will pay more attention to the function of AP000695.2 in tumor metastasis and tumor microenvironment regulation of LUAD. Furthermore, we will perform more *in vivo* and *in vitro* experiments to explore the function and the potential molecular mechanisms of AP000695.2 in tumor metastasis and tumor microenvironment regulation of LUAD.

## Conclusion

In conclusion, this study uncovers the biological function of AP000695.2 in LUAD for the first time. AP000695.2 was highly expressed in LUAD and associated with adverse clinical outcomes in LUAD patients, and AP000695.2 expression in LUAD is associated with pathologic stage, TNM stage, and residual tumor. Furthermore, AP000695.2 plays a significant role in regulating cell proliferation, cell migration, and DDP resistance. Our findings showed that AP000695.2 has the clinical potential to reverse DDP resistance and achieve better clinical outcomes in LUAD patients and may serve as a promising diagnostic and prognostic biomarker for LUAD.

## Data Availability

The original contributions presented in the study are included in the article/Supplementary Material, further inquiries can be directed to the corresponding authors.
